# Pelvic Fixation for Non-Ambulatory Patients with Neuromuscular Scoliosis Treated with Magnetically Controlled Growing Rods—A 4-Year Comparison of Two Different Fixation Methods

**DOI:** 10.3390/jcm13133845

**Published:** 2024-06-29

**Authors:** Annika Heuer, Sebastian Stuecker, Ralf Stuecker, Kiril Mladenov

**Affiliations:** 1 Department of Trauma and Orthopedic Surgery, University Medical Center Hamburg-Eppendorf (UKE), Martinistrasse 52, 20246 Hamburg, Germany; 2 Pediatric Orthopaedic Department, Altona Children’s Hospital, Bleickenallee 38, 22763 Hamburg, Germany; 3 Department of Orthopaedics, University Medical Center Hamburg-Eppendorf (UKE), Martinistrasse 52, 20246 Hamburg, Germany

**Keywords:** neuromuscular disease, scoliosis, orthopedic procedures, pediatrics, spinal disease, spine, pelvis, orthopedic fixation devices

## Abstract

**Background/Objectives**: This study aims to analyze the efficacy and safety of the two pelvic fixation systems, S-hooks (SH) and lumbar-sacral-alar-iliac (SAI) screws, when used in association with magnetically controlled growing rods (MCGRs) in non-ambulatory children with severe neuromuscular scoliosis (NMS). **Methods**: Forty-one patients were retrospectively examined and subdivided corresponding to ilium hook fixation or SAI screws. The major curve correction (%) and pelvic obliquity (PO) correction (%) were assessed utilizing scoliosis plain film radiographs over time. Complications and unplanned return to the operating room (UPROR) were recorded. Patient-specific pre- and postoperative values were included in a backward stepwise regression model to assess UPROR. **Results**: Mean age at index intervention was 9.4 years. Preoperative main curve was 81° and PO was 22°. 21 and 20 patients were categorized into the SH and SAI subgroups, respectively. Initial curve correction was significantly better in the SAI subgroup (63 vs. 42% in the SH, *p* = 0.045), while PO correction was equally good. Curve and PO correction were maintained throughout the follow-up period of 55 months. UPROR rate was 38% in the SH subgroup, and 5% in the SAI subgroup (*p* = 0.010). Regression analysis identified postoperative curve correction as predictive value for UPROR (*p* = 0.006). **Conclusions**: SAI screw fixation has a low UPROR rate and achieves superior curve correction. S-hooks are a viable option to correct PO and NMS in children with high operative risk to reduce operative time, but revision surgery is not uncommon.

## 1. Introduction

Early-onset scoliosis requires complex pediatric orthopedic management due to its progressive nature and the potential life-threatening risks it presents to children [1]. Surgical techniques are “growth-friendly” and aim at controlling the spinal curvature while facilitating continuous spinal and thoracic growth [2,3,4]. These methods are intended, among other objectives, to mitigate the effects that constrain the thoracic cavity, leading to restrictive lung disease, cardiovascular impairment, and respiratory failure [1,3]. Magnetically controlled growing rods (MCGRs) were first introduced by Takaso et al. and have gained popularity due to their non-invasive adjustability in an outpatient setting, significantly reducing the need for repeat surgeries and anesthesia, which are typically required every 6 months for traditional growing rod systems [2,3,5,6]. However, the efficacy is frequently undermined by complex issues such as severe pelvic tilt, associated hip disorders, and reduced bone quality [5].

In children with neuromuscular early-onset scoliosis (NMS), pelvic obliquity (PO) is a common and significant concern, often necessitating the extension of spinal fusion to the pelvis for stabilization, as significant PO can deteriorate sitting balance and overall health-related quality of life [7,8,9]. Multiple options exist for pelvic fixation in distraction-based growing rod systems, including pelvic hooks (S hooks) and various types of screw-based fixations (iliac, sacral-alar-iliac [SAI]) [10]. While the use of iliac and sacral-alar-iliac screws leads to a more robust construct, it lengthens surgery times and is technically more demanding [7,9]. Achieving a balance between clinical success and the reduction in surgical risks is paramount. S-hook fixation, while thought to be more prone to complications, reduces operative strain [10,11]. 

The objective of this single-center study is to investigate the effectiveness and safety profiles of S-hooks and SAI screws, when used in conjunction with MCGRs in treating NMS in non-ambulatory patients. This investigation will assess both techniques based on the incidence of additional unplanned surgical procedures, and pelvic obliquity and coronal curve correction using the Cobb angle. Addressing this unexplored area, our research aims to provide valuable insights and evidence-based guidance for clinical decision-making in the treatment of this complex condition.

## 2. Materials and Methods

### 2.1. Study Design and Participants

Retrospectively, we reviewed patients treated at our tertiary children’s hospital with a pediatric spine center led by a multidisciplinary team of pediatric orthopedic and pediatric neurosurgical specialists. All patients had previously been diagnosed with a neuromuscular disease and surgical treatment was indicated due to the severity or progression of their deformity. Furthermore, all patients were non-ambulatory and required pelvic fixation, either by SAI screw or S-hook fixation. The choice of pelvic fixation was at the discretion of the treating surgeon. We excluded patients with pure kyphotic or lordotic deformities and those who had been converted from other types of growth-friendly surgeries, such as VEPTR. 

This study was conducted and reported in accordance with the STROBE (Strengthening the Reporting of Observational Studies in Epidemiology) guidelines [12].

### 2.2. Data Collection

Clinical data were collected through an analysis of patients’ clinical reports, chart notes, surgical documents, and radiographic imaging results. This included detailed records of patient demographics, clinical presentations, surgical procedures, and follow-up assessments.

- Preoperative data: demographics [age, sex, body mass index, American Society of Anesthesiologists class, neuromuscular disease type], ambulatory status, and preoperative hemoglobin value.
- Surgical details: type of pelvic fixation, duration of surgery, intraoperative blood loss, use of intraoperative neurophysiological monitoring, use of cell salvage device, red blood cell (auto-) transfusion, use of rib cradles or thoracic screw placement, pedicle screw placement at the lumbar spine and sacrum, number of pedicle screws used, vancomycin use, hemoglobin low point, and postoperative hemoglobin value.
- Radiographic data: severity of NMS measured by Cobb angle, side of convexity, pelvic horizontal obliquity (PO), lumbar lordosis (LL), highest instrumented vertebra, and corresponding follow-up measurements at each time point.
- Postoperative data: In-hospital and complications throughout the follow-up period, follow-up duration, and BMI change.

### 2.3. Operative Technique

Spinal surgeries were performed in a prone position by a team comprising two senior surgeons. Intraoperative neurophysiological monitoring and red blood cell autotransfusion via cell salvage devices were utilized for all patients in the SAI subgroup.

**Figure 1:**
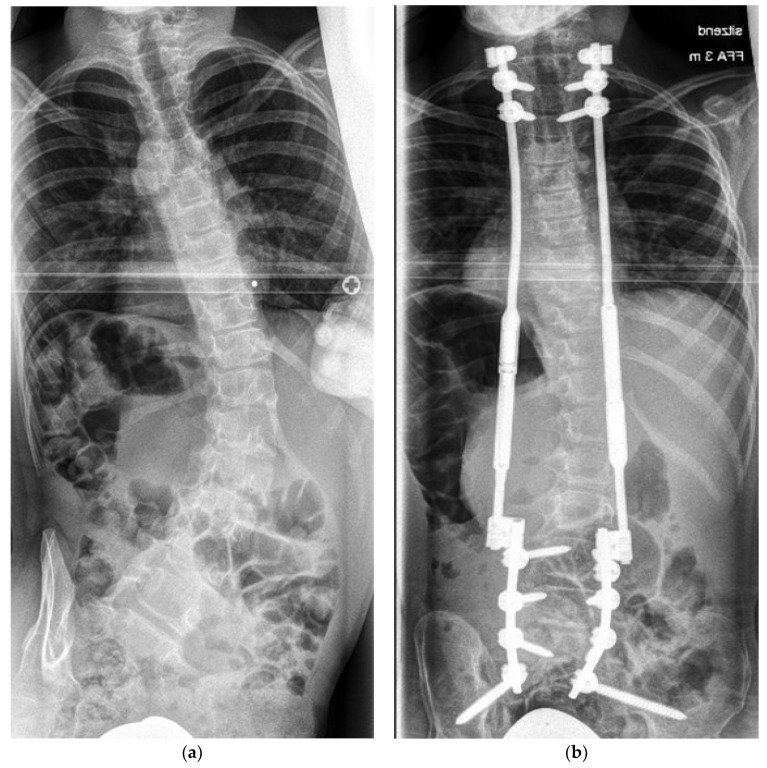
(**a**) Eleven-year-old boy with cerebral palsy and a coronal Cobb angle of 57°. (**b**) Instrumentation with pedicle screws L4 to S1 and pelvic fixation with SAI screws. Proximal fixation was achieved with pedicle screws in T2 and T3 with additional sublaminar bands attached to the second rib on both sides. A correction to 18° (Cobb) was achieved.

The standard approach in the SAI subgroup involved the placement of pedicle screws at L4, L5, and S1, complemented by SAI fixation in the original Sponseller technique [13]. Notably, anatomical variations required deviations from this approach, leading to the omission of S1 or L4 screws in some of the cases. Subsequently, a technique involving the unilateral anchoring of small rods to the screws, followed by rotation using a cantilever method, was employed to correct pelvic obliquity. The short rods were then connected to the MCGRs. Proximal stability was achieved using rib cradles in 26 patients, and a vertebral fixation involving a short fusion with pedicle screws alone or in combination with rib cradles was performed in 15 patients. The distraction construct extended from the thoracic spine to the ilium, as illustrated in Figure 1. 

**Figure 2:**
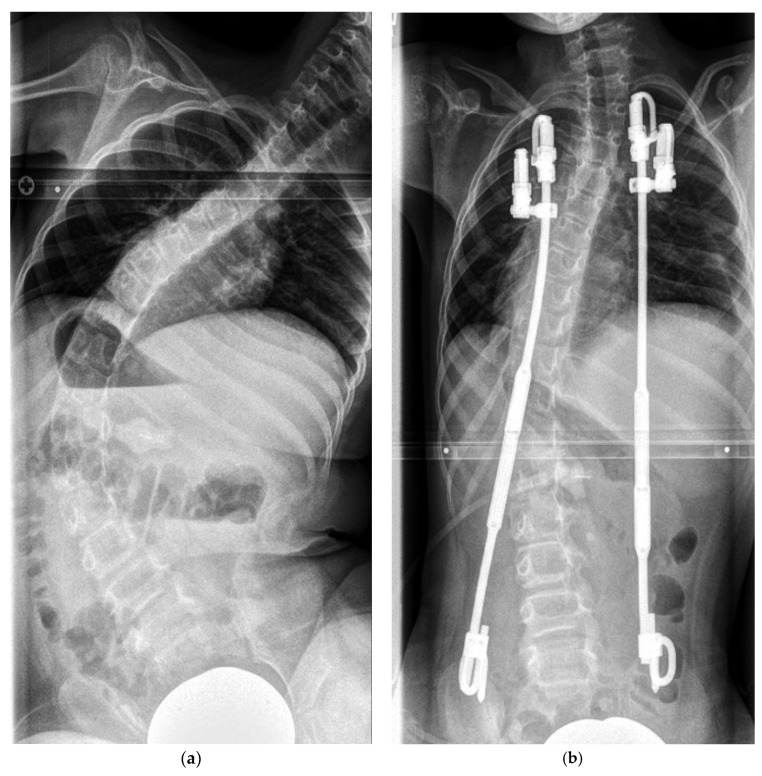
(**a**) Eight-year-old girl with cerebral palsy. Preoperative X-ray shows a coronal Cobb angle of 94°. (**b**) Instrumentation with a rib-to-pelvis construct utilizing S-hooks achieved a correction to 31° (Cobb).

For S-hook placements, following the procedure outlined by Smith et al. [14], a surgical incision ranging from 8 to 10 cm was made on either side, centered over the posterior superior iliac spine. This procedure enabled the attachment of S-hooks to MCGRs by means of a parallel connector. Typically, a 6 mm diameter MCGR was preferred to achieve a direct connection to S-hooks and proximal cradles spanning from the rib cage to the pelvis, as illustrated in Figure 2.

### 2.4. In-Hospital Care

Patients with NMS undergoing MCGR treatment were admitted to the ICU due to their underlying condition and comorbidities. Early postoperative mobilization was focused on a sitting position and initiated as tolerated by the patient on the day of the surgical intervention. Physical therapy was provided daily, focusing on promoting early mobilization and training of patient transfers. Laboratory data for both groups were obtained according to postoperative conditions, depending also on the use of a cell saver and blood loss. Postoperative X-rays in two planes were obtained on the third day after surgery, following successful mobilization to a sitting position. 

### 2.5. Follow-Up Care

In our outpatient clinic, follow-up appointments for MCGR distractions were scheduled every 4–6 months. Postoperative X-rays were taken at every second follow-up visit, which included distraction of the growing rods. Between these intervals, ultrasound was utilized to monitor the success of the distractions. 

X-rays were measured twice on each occasion by two orthopedic spine fellows. The measurement of preoperative and post-correction Cobb angles followed the methodology established by Cobb [15], while horizontal pelvic obliquity (PO) was assessed using the technique described by Osebold et al. [16]. To further minimize individual bias, measurements at two consecutive time points were grouped to form the categories: short-term (ST) follow-up (at a mean of 12 months), mid-term (MT) (at a mean of 24 months), and long-term (LT) follow-up (at a mean of 55 months). Curve correction rate and PO correction rate in percent refer to the preoperative baseline values [Curve correction rate (%)  =  [(pre Cobb − post Cobb)/pre Cobb] × 100]. Further, the delta, representing the magnitude and direction of change following postoperative correction, was employed to assess the statistical significance of variations at subsequent time points relative to the baseline established by the initial correction (postoperative). Lumbar lordosis was measured between L1 and S1. This structured approach allows for a comprehensive analysis of the treatment’s effectiveness over time, while reducing possible bias within each individual measurement.

### 2.6. Statistical Analysis

All analyses were performed using SPSS version 26 (IBM, Armonk, New York, NY, USA). Continuous variables are expressed as mean ± standard deviation (SD), and categorical variables are expressed as number and percentage (%). The Shapiro–Wilk test was used to test for normal distribution. The Student’s *t*-test for independent samples was employed for normally distributed continuous variables. The Mann–Whitney U test was used for non-normally distributed data, and the chi-square or Fisher’s exact test was applied to categorical variables. Repeated measures ANOVA with Greenhouse–Geisser correction and a post-hoc analysis with Bonferroni adjustment were used for normally distributed data, while non-normally distributed data underwent Friedman’s ANOVA and Wilcoxon post-hoc tests with Bonferroni adjustment. To assess prerequisites of regression analysis, Pearson correlation for continuous and Spearman correlation for nonparametric variables were applied, ensuring that correlations among predictors were less than 0.7. Both continuous and categorical variables were included in the logistic regression model. Predictors were log-transformed (Log base 10) for analysis if not normally distributed. The analysis utilized the stepwise backward logistic regression method, applying the maximum likelihood function to address nonlinearity in the influence of continuous variables. The model’s goodness of fit was assessed using Nagelkerke’s R2. Statistical significance was set at a 2-tailed *p*-value of <0.05, in accordance with accepted standards.

## 3. Results

### 3.1. Patient Demographics

We enrolled 41 consecutive patients, with a mean age of 9.5 ± 2 years at the time of surgical intervention. All patients underwent MCGR treatment. The cohort consisted of 54% (n = 22) females, with an average BMI Z-score of −3.3 ± 4. Due to their underlying neuromuscular conditions, all patients were non-ambulatory and presented with severe NMS, with a mean of 81 ± 19 degrees. NMS was most frequently attributable to infantile cerebral palsy level V (ICP V; 32%, n = 13) and spinal muscular atrophy type 2 (SMA 2; 30%, n = 12), as detailed in Table 1.

Patients were categorized based on the use of S-hooks or SAI screws for pelvic fixation, as shown in Table 1 and Table 2. Demographic characteristics revealed no significant difference between the two groups (Table 1). However, children in the SH subgroup had a statistically significantly worse mean scoliosis curve of 88.1 ± 13 degrees compared to the SAI subgroup (72.9 ± 19 degrees; *p* = 0.010). Preoperative pelvic obliquity (PO) and lumbar lordosis angle (LL) were similar between both groups, averaging 18.1 and 25 degrees respectively.

### 3.2. Operative Data

In our cohort, 21 (51%) patients were treated with a rib-to-pelvic construct using S-hooks (SH subgroup), while the remaining 20 (49%) underwent lumbosacral pedicle screw with additional SAI screw fixation (SAI subgroup). The average duration of surgery was significantly shorter in the SH subgroup, at 1 h and 54 min, compared to 3 h and 24 min in the SAI subgroup (*p* < 0.001). A positive correlation was noted between the amount of blood loss and surgical duration (*p* = 0.002). The lowest recorded hemoglobin (Hb) value post-surgery was 7.8 ± 1 g/dL in the SAI subgroup and 9.9 ± 1 g/dL in the SH subgroup (*p* < 0.001). By the first postoperative day, patients in the SH subgroup experienced an average decline of 1.7 g/dL in Hb levels without requiring blood transfusions. Conversely, children in the SAI subgroup had an average decrease of 3.9 g/dL Hb points, with 85% requiring either intra- or postoperative red blood cell (auto-)transfusions.

### 3.3. Radiological Follow-Up

The mean radiological follow-up period for this patient cohort was 55 ± 18 months. Although the SH subgroup maintained a more severe major curve compared to the SAI subgroup, the postoperative curve correction in the SH subgroup showed significant improvement to the preoperative baseline, with a mean correction of 51% (*p* < 0.001). The mean correction achieved in the SAI subgroup was significantly higher at 63% compared to the SH subgroup (*p* = 0.047), as illustrated in Table 3. This finding was independent of the preoperative Cobb values in the groups. Additionally, both groups had significant improvements in PO correction (*p* < 0.001), with the SH and SAI subgroups achieving mean improvements of 57% and 73%, respectively. However, the difference in PO improvement between the two groups was not statistically significant. Moreover, in both groups, LL increased in mean by 10° postoperatively (Table 4). 

Throughout follow-up, a decrease in PO correction was observed. In the SH subgroup, PO correction changed from 58% to 63% and lastly 59%. Meanwhile, the SAI subgroup’s PO correction decreased from 71% to 68% and lastly to 65%. This trend was not statistically significant within or between the groups.

**Figure 3:**
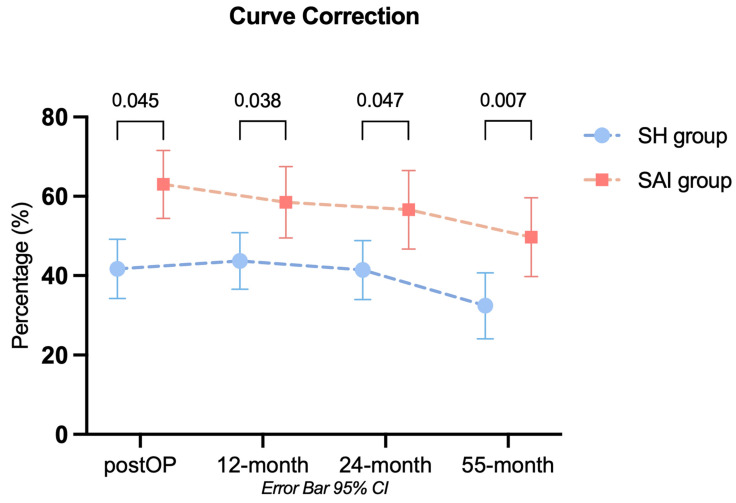
Shown are clustered error bar means of the variable “curve correction”. Each time point is compared to the baseline value to determine curve correction. The data show a significant difference between the curve correction achieved for the SH and SAI subgroups with superior results for the SAI subgroup throughout this study. Values with error bars depicting the mean with a 95% confidence interval.

Similarly, a gradual decrease in curve correction from the initial postoperative period was noted over time. In the SH subgroup, correction percentages decreased from 44% to 41% and 32%. For the SAI subgroup, the correction percentages changed from 59% to 57% and 50%. This trend was not statistically significant within or between the groups. Achieved correction compared to the preoperative values was significantly superior in the SAI subgroup for all time points (Figure 3) but no significant interaction between time points and groups could be observed regarding the direction of change following postoperative correction. Therefore, after the initial postoperative correction, the SAI subgroup showed no further significant improvements compared to the SH subgroup.

While no between-group effects were observed for LL, a significant linear trend towards an increasing LL was shown over time across all measurements in both groups (*p* = 0.049). 

### 3.4. Mechanical Complications

Complications arising from the MCGR procedure were categorized into major complications necessitating unplanned surgical intervention and minor complications that did not require additional invasive therapy and were resolved without further action or management during the next scheduled routine MCGR device exchange. Throughout this study, we encountered nine (22%) major mechanical complications necessitating surgical revision. Unplanned return to the operating room (UPROR) occurred significantly more frequently in the SH subgroup (38%) compared to the SAI subgroup (5%; *p* = 0.010). In the SAI subgroup, only one patient was confronted with an UPROR due to a deep wound infection, which involved implant removal and re-implantation following successful treatment. Additionally, two instances of hardware malfunction (minor) coincided closely with scheduled hardware replacements, thereby obviating the need for additional interventions. Conversely, the SH subgroup experienced seven UPROR events due to S-hook dislocations causing loss of correction. Among these, two patients later underwent further UPROR due to a cradle dislocation and a late infection. Another patient underwent implant removal and halo traction for a low-grade infection before the final fusion procedure. Additionally, in one case, a dislocated cradle and in another case, a dislocated S-hook were addressed during the next scheduled implant change (minor).

The necessity for revision surgery did not significantly affect the degree of curve correction at any observed time point, either within or between the subgroups. Further analysis using a backward stepwise regression to identify predictors of revision—including variables such as age at surgical intervention, sex, segment length of the curve, preoperative PO, postoperative PO correction, initial curve degree, and the degree of postoperative curve correction—resulted in a model with a Nagelkerke R square of 0.632, accurately classifying between 87.8% and 90.2% of cases. Within this model, the degree of postoperative curve correction was the only significant predictor (B = −0.122, *p* = 0.006, Exp(B) = 0.885).

## 4. Discussion

In this longitudinal study spanning 55 months, we observed 41 non-ambulatory children with NMS treated with MCGRs. Our main study findings were as follows:

(A) Horizontal PO correction was successfully achieved and maintained over the mean duration of 55 months. This result was independent of the pelvic fixation method.
(B) The S-hook subgroup presented with more severe scoliosis at baseline and experienced a significant higher rate of UPROR.
(C) SAI screw fixation led to a substantially greater initial correction of scoliosis, which was effectively maintained throughout the treatment period.

Whilst previous studies supported the notion that MCGRs successfully control curve progression and aid spinal development in early onset scoliosis (EOS), they are not without risk [17]. In our study, 22% of patients had an UPROR, which is at the lower margin of previously reported rates for EOS treated by MCGR constructs. Initial curve correction was 57% postoperatively, which decreased slightly to 49% at the one-year follow-up. Overall, correction was maintained at 49% at two years and further decreased to 41% until the four-year follow-up, which is in line with the current literature. Thakar et al. analyzed the complication profile in early-onset scoliosis in a systematic review including fifteen studies and 336 patients (including 66 NMS) with a mean age of 7.9 years and a 1-year follow-up. While correction and retention were successful, they found a 45% cumulative non-medical complication rate, coinciding with 33% of unplanned revisions including 12% anchor pull-out, 12% implant failure (breakage of the actuator pin or rod slippage), and 11% rod breakage [17]. The authors further concluded that the articles did not report implant pull-out according to the fixation type (hooks, screws, or hybrids) and therefore no evidence-based superiority could be determined [17]. Grabala et al. included 161 children (including 51 NMS) with a mean age of 7 years in a multicenter approach with two-year follow-up. Average curve correction was 50% throughout the lengthening period and increased to 70% after the final posterior spinal fusion procedure. Twenty percent of patients required an unplanned revision surgery, with the highest overall complication rate in the NMS subgroup. Complications included five distraction failures, four rod breakages, four infections, three anchor pull-outs, three pin fractures, and two cases of proximal junction kyphosis. Whilst segmental screw fixation cranially and caudally was mentioned, no further information is available regarding the specific techniques used [18]. Welborn et al. retrospectively analyzed 106 patients with severe scoliosis (50–114°) and a 3.5-year follow-up. Their findings included an UPROR in 26% of cases and association of UPROR with male gender [19]. Urbanski et al. treated 47 patients (including 10 NMS) with a mean age of 8.8 years either with a single- or dual-rod construct (24/23 patients) and described an immediate scoliosis curve correction of 32.4% with an overall correction of 27.5% at their one-year follow-up. They found that patients with NMS had the best curve correction, which was corrected from a preoperative mean Cobb of 65° to 51° postoperatively. Further, 28% of UPRORs including implant failures (17%), wound infections (4%), and distraction failure were reported. The construct used caudal segmental screw fixation without further description [20]. Lebel et al. included 47 patients (17 NMS) in a multicenter study with a 4-year follow-up period and mean age of 9.2 years. Surgical procedures were carried out including the use of hooks, pedicle screws, or both, with a distal screw foundation not further described. The initial mean curve of 70° Cobb was reduced to 40° following the index surgery. After a mean of 4.2 years, the deformity increased to 53°. Within the NMS group, a mean preoperative curve of 76° was present and corrected to 44° initially with an increase to 57° during follow-up. Sixty-six percent of patients in this cohort had at least one complication, with forty-four percent of UPROR. Within the NMS group, 41% underwent UPROR with 18% due to infections, 12% proximal foundation failure, and 12% rod breakage [21]. 

The importance of restoring sagittal alignment in EOS with MCGRs is increasingly recognized. In a study of 37 ambulatory EOS patients (mean age 8.5 years), 70% achieved balanced constructs postoperatively. Significant reductions were observed in maximal thoracic kyphosis and maximal lumbar lordosis, while the number of vertebrae contributing to lumbar lordosis increased by two vertebrae on average. This shifted the thoraco-lumbar inflection point and kyphosis apex cranially. Ilharreborde et al. concluded that MCGR insertion flattened the spine and altered overall spinal harmony, with a cranial shift and lengthening of lumbar lordosis [22]. The Harms study group analyzed the influence of spinal fusion for scoliosis in 84 non-ambulatory patients with CP, with a mean of 16 levels fused and 94% including fusion to the sacrum. They found a significant increase in postoperative LL and identified secondary postoperative lumbar hyperlordosis (>60°) as a risk factor for worsening postoperative hip status (subluxation/dislocation). The authors concluded that hyperlordosis and loss of protective lumbopelvic motion (fusion) caused anterior pelvic tilt and functional acetabular retroversion [23]. In our clinical experience, we observed two patients treated with SAI screw fusion of L4-S1 at a young age who developed increasing lumbosacral lordosis (L4-S1) and consecutive anterior pelvic tilt. This likely resulted from a substantial remaining growth potential, restricted posterior spinal growth through fusion, and continued anterior vertebral body growth, leading to exaggerated lumbar lordosis over time [24,25,26]. The crankshaft phenomenon, referring to deformity progression after posterior spinal fusion due to continued anterior spinal growth, was first described by Dubousset in 1973 and has been extensively researched. However, lumbosacral changes after pelvic fusion have yet to be explored [24,27]. While growing rod constructs were developed to allow maximum spinal growth, it remains unclear how spinopelvic fusion in very young children affects the sagittal profile. Conversely, a study using a canine model demonstrated that pedicle screws and stiff posterior instrumentation inhibited lordosis increase secondary to persistent anterior growth after non-instrumented posterior fusion. Pedicle screw instrumentation acted as mechanical epiphysiodesis, arresting vertebral body lengthening and preventing hyperlordosis [28]. While our work supports the preferable use of SAI screws, more research is needed to analyze potential negative aspects on the sagittal profile of SAI screws in very young patients.

**Figure 4:**
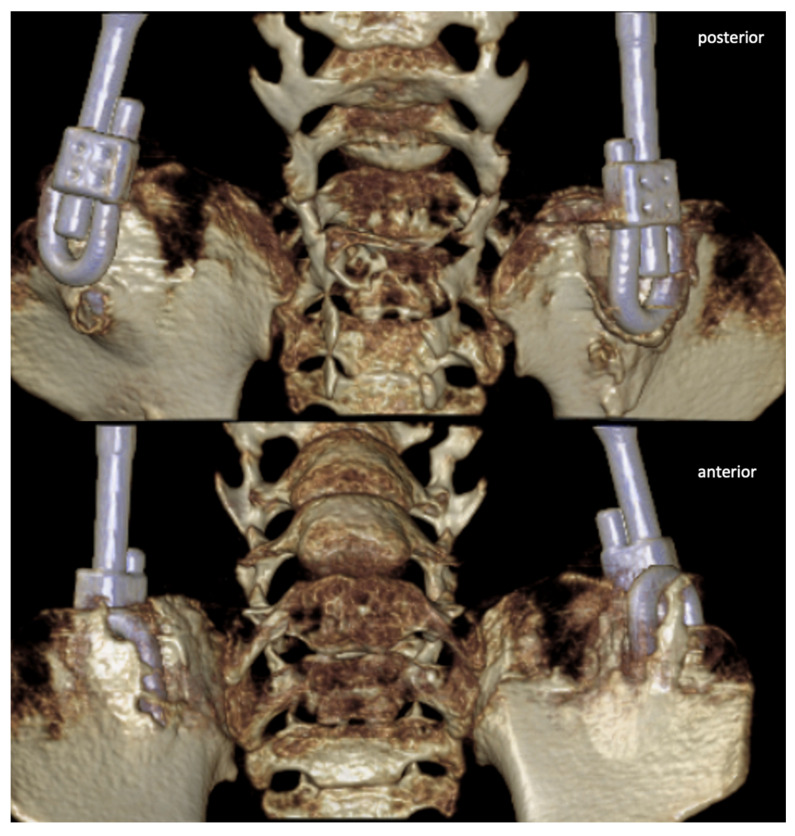
CT graphic 3D reconstruction showing heterotopic ossification around the S-hook. (**Top**) picture shows a posterior view, whilst the (**bottom**) picture depicts an anterior view.

While a variety of surgical techniques and systems exists, the specific treatment choice is largely based on the surgeons’ personal experience due to a low level of preexisting evidence and the highly individual characteristics of patients and deformities [29]. S-hook utilization allows for a more lateral positioning of the MCGRs, positioning the construct further away from the apex region of the main scoliosis curve avoiding a crossover of the midline at the region of the apex with the convex rod. We aim to avoid or reduce contact of the construct with the bony structures of the spine itself, as the resulting mechanical stimulation from the rods, coupled with their rigid fixation, facilitates an environment conductive to bone growth and auto fusion at these contact areas [30]. This is the reason most surgeons prefer the use of S-hooks for greater deformities and explains the fact that curve magnitude in the SAI subgroup were significantly smaller than in the SH subgroup. An example for heterotopic bone formation is shown in Figure 4. This case was triggered by persistent mechanical irritation through micro-motion and microtrauma due to the less rigid connection achievable through S-hook fixation.

So far, few studies have focused on caudal and especially pelvic anchor techniques used for MCGR treatment and usually only compare lumbar versus pelvic constructs rather than two separate types of pelvic anchoring [31,32,33,34]. For growth-friendly surgery, fixation to the ilium has proved to be superior to sacral fixation, and further, SAI screws have been shown to be favorable to ilium-only screws [31,32,33,35,36]. In particular, a greater correction in the main curve and pelvic obliquity was shown in patients with non-ambulatory NMS [36,37]. Brooks et al. compared the pelvic fixation of 38 EOS patients (26 NMS) to the ilium (screws or L-rods) and/or sacrum in different growing rod constructs and concluded that anchors to the ilium significantly improved the major curve and PO compared to sacrum-only constructs [36]. Schur et al. included 153 EOS patients (95 NMS) receiving either SAI screws or iliac screws (42) as pelvic fixation method and patients with S-hook fixation (111) in a multicenter study combined with a VEPTR construct. Most common complications were device migration (13), implant failure (8), and prominence (4) for the S-hook fixation whilst for the screw group they were implant failure (3), prominence (2), and device migration (1). They found screw constructs to achieve better PO correction than S hooks [37].

In our patients, 21 constructs were anchored with an S-hook fixation whilst the other 20 constructs utilized SAI screws combined with lower lumbar spine and S1 pedicle screws. Our data showed successful PO correction in both groups as well as a good long-term control of the achieved PO with a mean of 6.2 degrees at the 4-year follow-up. With this outcome, we stayed well below the threshold of 7 degrees suggested by Matsumoto et al. for NMS patients who have undergone scoliosis surgery. They identified this threshold as crucial for the improvement of health-related quality of life in both children and their caregivers [8]. However, a significantly greater initial postoperative curve correction was achieved in the SAI subgroup. This lesser curve correction achieved with S-hooks and the greater preoperative baseline curve are the reason for a significant lower mean Cobb angle in the SH subgroup throughout the follow-up. The superiority of the SAI subgroup was maintained at all following time points, whilst no further additional improvement (delta of change between the time points) was achieved with subsequent distractions compared to the SH subgroup. Therefore, SAI screw fixation showed a significantly higher capacity of curve correction at the initial surgery compared to S-hook fixation, whilst both techniques were able to maintain correction throughout the follow-up period.

In a previous study from our institute on 20 patients with EOS treated with bilateral rib-to-ilium VEPTR constructs complications related to S-hooks were observed in six cases (30%) [38]. In this most recent work with an MCGR construct, revision surgeries due to complications were significantly more common in the SH subgroup (38%) compared to the SAI subgroup (5%). In the SAI subgroup, the only patient requiring UPROR did so due to a deep wound infection. Conversely, in the SH subgroup, seven S-hook dislocations required surgical intervention whilst one patient underwent surgery due to a low-grade infection. The degree of the achieved postoperative curve correction was identified as the only predictive variable. Each degree of postoperative curve correction showed a protective effect against the likelihood of requiring revision surgery, reducing the odds of an UPROR by 11.5%. As most complications were due to mechanical problems in the S-hook group, lesser curve correction might lead to increased contact stress on the construct that primes for S-hook-related complications.

Ramirez et al. reported on 65 children (26 NMS) treated with a VEPTR–S-hook construct in a multicenter setting with a 46-month follow-up. Fifty percent of their patients had S-hook-related complications with the most common being lateral or medial sliding of the hook whilst they documented a 9% infection rate. In addition, they reported an association of complication rate and use of end-to-end connectors instead of domino connectors regarding ambulatory status, especially when developing a postoperative crouched gait and surgical time over three hours [12].

Another consideration in choosing pelvic fixation is the type of strain put on patients during the initial surgical intervention. Lumbosacral pedicle screw fixation significantly increases surgical time and blood loss. Our patient cohort of non-ambulatory NMS patients is especially vulnerable, and surgical duration has to be carefully weighed against a potential risk for UPROR or less achievable correction. We suggest considering S-hook fixation in “high risk” patients with multiple comorbidities or as alternative when a high bony spine and rod contact overlap is not preventable. As shown in our study, UPRORs due to anchor complications need to be anticipated in this patient cohort. Non-ambulatory patients with NMS without anatomic anomalies, mechanic obstacles, or anesthesia contraindications can achieve higher coronal curve correction and less UPROR by a SAI-based pelvic fixation.

### Limitations

Cobb angle correction may be influenced by different factors such as curve flexibility, stability of proximal anchors, and bone quality. These factors could not be evaluated in this study and may represent a major limitation. This study’s sample size was limited due to the specific sub-analysis of only NMS with non-ambulatory status versus EOS in children with different level of mobility and further limited to one specific growing rod construct, a valuable subgroup that was not assessed in this manner before. As the sagittal profile in this patient cohort could not be measured consistently, it was excluded from the radiographic evaluation. This aspect will be addressed comprehensively in a future study.

## 5. Conclusions

In non-ambulatory patients with NMS treated with MCGRs, both S-hook and SAI lumbar pedicle screw fixations to the pelvis successfully improved and maintained PO over the 55-month follow-up period. However, lumbosacral fixation using SAI screws not only significantly reduces the risk of UPROR but also achieves a higher initial curve correction that was maintained throughout the follow-up period. Conversely, while the use of S-hooks shortens surgical time, a higher risk for revision surgery should be anticipated.

## Figures and Tables

**Table 1:** Shown are demographic and operative characteristics of the two groups.

Variables	All	SH	SAI	*p*-Value
Female (%, n)	54% (22)	66.7% (14)	40% (8)	0.087
Age (years; mean ± SD)	9.4 ± 1.9	9.3 ± 2	9.5 ± 1.8	0.734
BMI (kg/mL; mean ± SD)	14.8 ± 3.7	15.3 ± 4	14.2 ± 3.1	0.338
BMI Z-score (mean ± SD)	−3.3 ± 3.6	−2.8 ± 3.7	−3.7 ± 3.5	0.446
Neuromuscular disease				0.325
ICP V (%, n)	31.7% (13)	38.1% (8)	25% (5)	
SMA 2 (%, n)	29.3% (12)	28.6% (6)	30% (6)	
Myopathy (%, n)	12.2% (5)	9.5% (2)	15% (3)	
AMC (%, n)	7.3% (3)	1, 4.8% (1)	10% (2)	
MMC (%, n)	4.9% (2)	9.5% (2)	0% (0)	
Chromosomal (%, n)	4.9% (2)	0% (0)	10% (2)	
Flaccid paraplegia (%, n)	4.9% (2)	9.5% (2)	0% (0)	
Rett syndrome (%, n)	2.4% (1)	0% (0)	5% (1)	
MCD1A (%, n)	2.4% (1)	0% (0)	5% (1)	
Operative time (min, mean ± SD)	159 ± 58	116 ± 31	204 ± 43	**<0.001**
Unplanned revision surgery (%, n)	22% (9)	38.1% (8)	5% (1)	**0.010**
Blood (Re-)transfusion (%, n)	41.5% (17)	0% (0)	85% (17)	**<0.001**
Hb low point (mean ± SD)	8.9 ± 1.5	9.9 ± 1	7.8 ± 1.2	**<0.001**
Hb change pre- to postoperative (mean ± SD)	−2.8 ± 2.1	−1.7 ± 1.2	−3.9 ± 2.3	**<0.001**
Follow-up (month, mean ± SD)	54.6 ± 17.5	59.3 ± 19.6	49.7 ± 13.8	0.079

SH subgroup—S-hook fixation cohort; SAI subgroup—sacral-alar-iliac screw fixation cohort; BMI—body mass index; ICP V—infantile cerebral palsy level V; SMA 2—spinal muscular atrophy type 2; AMC—arthrogryposis multiplex congenita; MMC—myelomeningocele; MCD1A—congenital muscular dystrophy type 1A; Hb—hemoglobin; SD—standard deviation; min—minutes; ()—number of patients; bold print—significant, blue indicates the SH subgroup and orange indicates the SAI subgroup.

**Table 2:** Patient characteristics in the two study groups regarding coronal and sagittal plane spinal deformity degree (Cobb angle) and horizontal pelvic obliquity (degree). All patients initially presented with a severe degree of scoliosis.

SH Subgroup					SAI Subgroup		
Patient No.	Convex Side	Curve Degree	Horizontal Pelvic Obliquity	Patient No.	Convex Side	Curve Degree	Horizontal Pelvic Obliquity
*1*	Left	109	26	*1*	Right	68	17
*2*	Left	84	5	*2*	Left	60	19
*3*	Left	87	15	*3*	Left	53	10
*4*	Right	96	8	*4*	Right	90	7
*5*	Left	94	9	*5*	Right	62	22
*6*	Right	68	11	*6*	Left	100	30
*7*	Left	85	60	*7*	Right	72	12
*8*	Right	81	13	*8*	Right	57	32
*9*	Left	102	34	*9*	Right	96	14
*10*	Right	81	33	*10*	Right	70	30
*11*	Right	110	32	*11*	Right	109	37
*12*	Left	105	25	*12*	Right	35	18
*13*	Left	82	15	*13*	Left	56	16
*14*	Right	98	25	*14*	Left	85	35
*15*	Left	88	8	*15*	Right	62	7
*16*	Left	57	10	*16*	Left	55	27
*17*	Right	68	11	*17*	Left	70	20
*18*	Left	83	34	*18*	Left	78	30
*19*	Right	81	20	*19*	Right	98	51
*20*	Left	98	20	*20*	Right	82	26
*21*	Left	116	27				

SH group—S-hook fixation cohort; SAI subgroup—sacral-alar-iliac screw fixation cohort, blue indicates the SH subgroup and orange indicates the SAI subgroup.

**Table 3:** Patient characteristics regarding main curve and PO. Further, degree of curve correction for short-term, mid-term, and long-term time points are provided. Information regarding growth development is analyzed by BMI, Z-score, and direction of change from the preoperative measurement.

Variable	All Subjects		SH Subgroup		SAI Subgroup		*p*-Value
	Mean	±SD	Mean	±SD	Mean	±SD	
**Preoperatively**							
Main curve	81.2	18.8	89.2	14.9	72.9	19.0	**0.008**
Pelvic obliquity	22.0	12.1	21.0	13.1	23.0	11.2	0.603
**Postoperatively**							
Main curve	36.1	19.7	44.2	19.3	27.7	16.7	**0.007**
Curve correction (%)	56.6	18.6	41.7	16.4	63.0	18.3	**0.045**
Pelvic obliquity	6.4	5.8	7.43	5.6	5.4	5.8	0.266
**Short Term (ST) Follow-up**							
Main curve	41.1	19.7	51.1	15.2	30.7	17.5	**0.001**
Curve correction (%)	48.9	20.1	43.7	15.6	58.5	19.2	**0.038**
Pelvic obliquity	6.4	6.3	7.2	5.0	6.0	5.6	0.494
**Mid Term (MT) Follow-up**							
Main curve	72.4	15.8	51.5	14.1	32.6	20.3	**0.003**
Curve correction (%)	48.8	20.1	41.4	16.4	56.6	21.1	**0.047**
Pelvic obliquity	5.5	4.4	6.7	5.0	6.1	3.8	0.648
**Long Term (LT) Follow-up**							
Main curve	48.2	19.8	59.4	14.9	38.2	17.9	**<0.001**
Curve correction (%)	40.9	21.4	32.4	18.2	49.7	21.2	**0.007**
Pelvic obliquity	6.2	3.3	6.2	3.5	6.2	3.1	1.00
**Growth at Last Follow-up**							
Last BMI (kg/mL)	16.1	4.2	16.1	4.0	16.1	4.5	0.990
Last BMI Z-score	−2.9	4.1	−2.1	2.6	−3.7	5.2	0.241
Z-score change from preop	0.4	3.9	0.7	3.9	0.04	3.9	0.584

SH subgroup—S-hook fixation cohort; SAI subgroup—Sacral-alar-iliac screw fixation cohort; Bold print is significant, blue indicates the SH subgroup and orange indicates the SAI subgroup.

**Table 4:** Patient characteristics regarding lumbar lordosis. Further, information regarding direction of change is included.

Variable	All Subjects		SH Subgroup		SAI Subgroup		*p*-Value
	Mean	±SD	Mean	±SD	Mean	±SD	
**Preoperatively**							
LL	25	29	25	25.0	26	33.0	0.754
**Postoperatively**							
LL	36	15	34	16.0	35	14.0	0.855
Delta PräOP-PostOP	10	25	11.0	20.0	11.0	31.0	0.933
**Short Term (ST) Follow-up**							
LL	35	14	34	16.0	36	14.0	0.865
Delta PostOP-ST	−0.5	5	−2	6.0	0.9	4.0	0.215
**Mid Term (MT) Follow-up**							
LL	35	9	34	13.0	36	14.0	0.865
Delta ST-MT	−0.5	16	−0.3	8.0	0.8	11.0	0.098
**Long Term (LT) Follow-up**							
LL	36	18	34	19.0	38	17.0	0.348
Delta MT-LT	0.7	11	0.1	12.0	2.4	10.0	0.705

SH subgroup—S-hook fixation cohort; SAI subgroup—Sacral-alar-iliac screw fixation cohort; LL—lumbar lordosis; Bold print is significant, blue indicates the SH subgroup and orange indicates the SAI subgroup.

## Data Availability

The datasets generated during and/or analyzed during the current study are not publicly available but are available from the corresponding author on reasonable request.
